# Demographic trends of motor neurone disease-associated mortality from 1999-2020 in the United States

**DOI:** 10.3310/nihropenres.13786.2

**Published:** 2025-08-26

**Authors:** Delaram Imantalab, Balamrit Singh Sokhal, Sowmya Prasanna Kumar Menon, Seema Kalra, Sara Muller, Christian Mallen

**Affiliations:** 1 Keele University School of Medicine, Keele, England, UK; 2 University Hospitals of North Midlands NHS Trust, Stoke-on-Trent, England, UK

**Keywords:** Motor Neurone Disease; Mortality; Outcomes; Epidemiology.

## Abstract

**Introduction:**

Motor Neurone Disease (MND) is a neurodegenerative condition affecting motor neurons in spinal cord and brainstem, leading to a reduced life expectancy. This study describes demographic trends in MND-associated mortality in the United States over a 20-year period.

**Methods:**

Data was extracted from the Centers for Disease Control and Prevention Wide-Ranging OnLine Data for Epidemiologic Research underlying cause of death database. All death certificates from 1999–2020 with MND (G12.2) recorded as the cause of mortality were extracted. Annual MND-associated crude mortality rates (CMR) and age-adjusted mortality rates (AAMR) per 100,000 persons with 95% confidence intervals (CI) were calculated. Joinpont regression was used to calculate the annual trends in MND-associated mortality by calculating the annual percentage change.

**Results:**

Between 1999 to 2020, there were a total 140,945 MND-associated deaths. Overall AAMR was 1.9 per 100,000 persons (95% CI 1.9-1.9). Male sex had a consistently higher AAMR (2.3 per 100,000 95% CI 2.3-2.3) than female sex (1.6 per 100,000 95% CI 1.5-1.6) across the study period. White patients had higher AAMR (2.1 per 100,000 95% CI 2.0-2.1) than Black/African Americans (1.1 per 100,000 95% CI 1.0-1.1), American Indians/Alaska Natives (0.8 per 100,000 95% CI 0.7-0.9), Asians/Pacific Islanders (0.8 per 100,000 95% CI 0.7-0.9). The 3 US States with the highest AAMR were Vermont, followed by Minnesota and Maine.

**Conclusions:**

There are a significant number of MND-associated deaths annually in the United States. The knowledge of these trends facilitates the design of appropriate services in areas of higher need, allowing for the introduction of pathways that support more suitable care and enhanced quality of life.

## Plain Language Summary

Motor Neurone Disease (MND) is a neurological condition affecting motor neurons in spinal cord and brainstem. MND has a poor life expectancy. We aimed to describe the demographic trends in deaths from MND over a 20 year period. Deaths associated with MND were extracted from a national database from 1999–2020. From 140,595 deaths we found that male sex and white race were characteristics associated with the highest mortality rates when age was taken into account. Knowledge of these trends facilitates the design of appropriate services in areas of higher need, allowing for the introduction of pathways that support more suitable care and enhanced quality of life.

## Introduction

Motor Neurone Disease (MND) is a rapidly progressing neurological disease characterised by the degeneration of upper and lower motor neurones and thereby deterioration of motor function ^1^. MND has several subtypes including Amyotrophic Lateral Sclerosis (ALS), progressive bulbar palsy, progressive muscular atrophy, primary lateral sclerosis, spinal muscular atrophy and post-polio syndrome ^1, 2^.

The incidence of MND in the United States (US) is approximately 2 in every 100,000 of the population annually. Mortality, as well as incidence, is most common in patients aged between 55 and 75 ^3– 5^. Recent epidemiological trends suggest an increased prevalence and incidence of cases of MND over the time period of 2015 to 2019 which is similar to trends reported in Europe. In the US, the average length of time from the point of diagnosis to death is three years ^3^. The most common cause of death secondary to MND is neuromuscular respiratory failure, which typically occurs between two and five years following the onset of the disease ^6^. Factors associated with improved survival included a younger age of onset and attending a regional MND clinic ^7^. MND is most common in white patients, therefore the majority of deaths occur in white patients ^8^. A population-based study in England, including mortality data from 1993–2010, concluded that home deaths were more frequent than deaths occurring at a hospice for patients with MND (27.1% versus 11.2%) ^1^.

Data are limited about the demographic trends in mortality from MND in the US. Knowledge of these trends is fundamental to allow healthcare providers to ascertain the factors associated with improved survival for MND patients and identify populations facing a disparity of healthcare provision. Therefore, the aim of this study was to use national death certificate data to explore demographic trends in MND-associated mortality.

## Methods

### Patient and Public Involvement

This study did not have patient or public involvement.

Data were extracted from the Centers for Disease Control and Prevention Wide-Ranging OnLine Data for Epidemiologic Research (CDC-WONDER) underlying cause of death database. CDC-WONDER contains details on the cause of death from death certificates from the 50 US states and the District of Columbia ^9^. More than 99% of US deaths are recorded on CDC-WONDER ^9^. Several studies have used this database to study a variety of conditions ^10– 14^.

Using the International Classification of Diseases-Tenth Revision (ICD-10), all death certificates from 1999–2020 with MND (G12.2) recorded as a cause of mortality were extracted. Data such as year, death count, population size, demographic data such as age, sex, race, Hispanic status, geographic data such as census region and state, and other death certificate data such as place of death were also extracted. Geographical data included urbanisation levels including metropolitan (large central metropolitan (1 million or more population with a high-population density), large fringe metropolitan (1 million or more population with a high population density in counties that do not qualify as large central metropolitan), medium metropolitan (counties with populations of between 250,000–999,999 with a high population density), small metropolitan (counties with populations less than 250,000 with a high population density)), and non-metropolitan (micropolitan (counties with populations around 10,000–50,000 population) and noncore (counties that do not qualify as micropolitan)) ^15^.

Annual MND-associated crude mortality rates (CMR) and age-adjusted mortality rates (AAMR) per 100,000 live persons in the population were calculated. To calculate CMR, the number of MND-associated deaths was divided by the population in the given year. Results were calculated with 95% confidence intervals (CI). AAMR was calculated by standardizing the MND-associated deaths to 1999 US population with 95% CI ^16^. Joinpont regression was used to calculate the annual trends in MND-associated mortality by calculating the annual percentage change (APC). Joinpoint regression identifies significant differences in AAMR over time using log-linear regression models for temporal variations. Joinpoint regression software was used for APC and trend calculations ^17^.

CDC-WONDER is an publicly available, anonymised dataset, and therefore did not require ethical approval from an institutional review board ^9^. The STrengthening the Reporting of Observational studies in Epidemiology (STROBE) guidelines were used to report this study ^18^.

## Results

### Annual trends for MND-associated mortality

**Figure 1.**
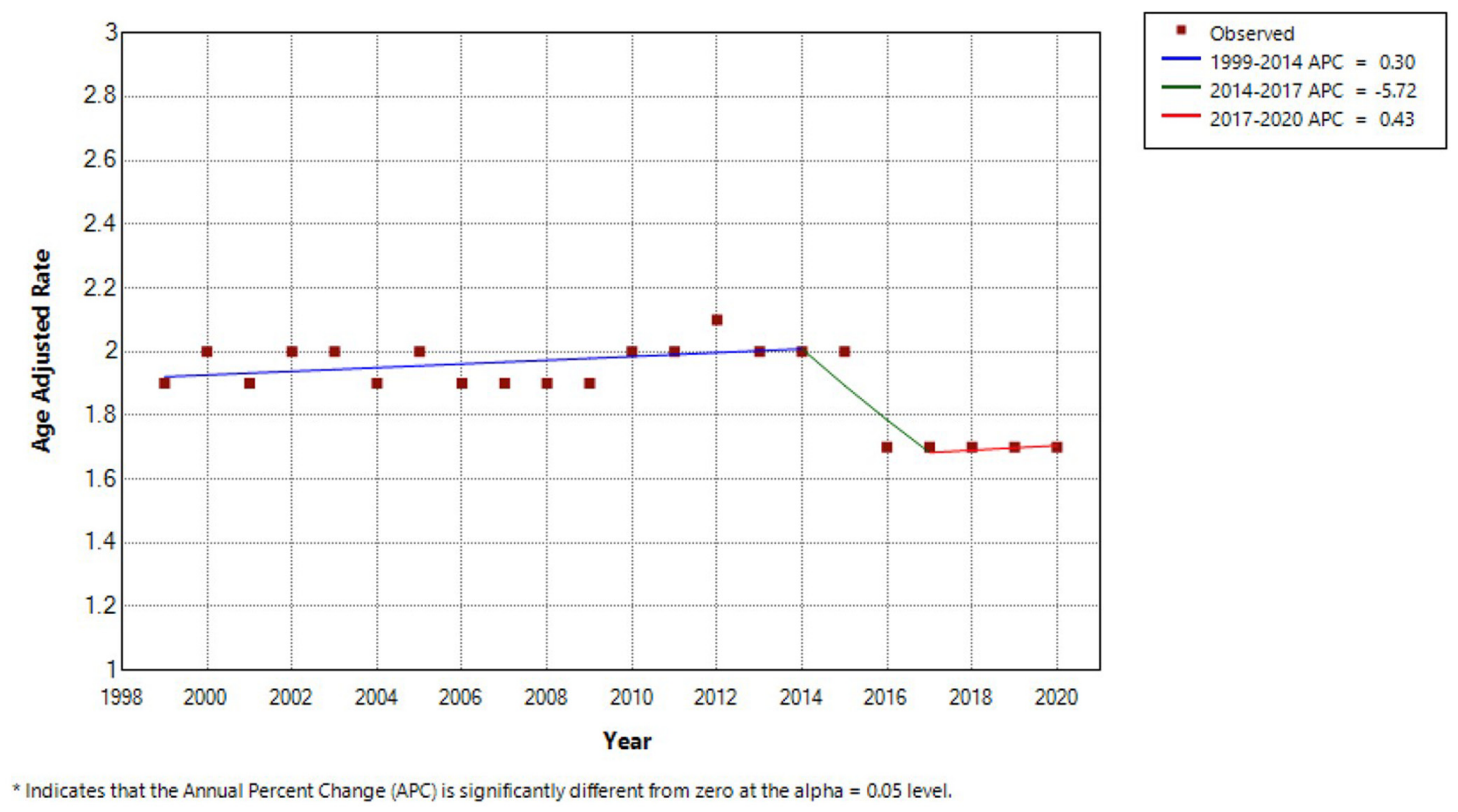
Joinpoint regression analysis by year.

Between 1999 to 2020, there were a total 140,945 MND-associated deaths. Overall, CMR was 2.1 per 100,000 persons (95% CI 2.1-2.1) and AAMR was 1.9 per 100,000 persons (95% CI 1.9-1.9). CMR was 1.8 per 100,000 in 1999 (95% CI 1.8-1.9) and 2.2 per 100,000 in 2020 (95% CI 2.2-2.2). AAMR was 1.9 per 100,000 in 1999 (95% CI 1.8-1.9) and 1.7 per 100,000 in 2020 (95% CI 1.7-1.8). Using Joinpoint regression analysis, the APC in AAMR was 0.30 (95% CI -2.81-5.94) from 1999–2014, -5.72 (95% CI -8.70-3.62) from 2014–2017 and 0.43 (95% CI -4.20-7.23) from 2017–2020 ( Table 1 and Figure 1).

**Table 1.** Demographic trends in mortality by year.

		1999	2000	2001	2002	2003	2004	2005	2006	2007	2008	2009	2010	2011	2012	2013	2014	2015	2016	2017	2018	2019	2020	1999–2020
**Overall ** **data**	**Number of** ** MND-related ** **deaths**	5,127	5,482	5,398	5,723	5,868	5,663	5,872	5,954	6,073	6,197	6,373	6,648	6,849	7,207	7,044	7,271	7,403	6,702	6,862	6,915	7,042	7,272	140,945
	**Population**	279,040, 168	281,421, 906	284,968, 955	287,625, 193	290,107, 933	292,805, 298	295,516, 599	298, 379, 912	301,231, 207	304, 093, 966	306, 771, 529	308, 745, 538	311,591, 917	313,914, 040	316, 128, 839	318,857, 056	321, 418, 820	323, 127, 513	325, 719, 178	327, 167, 434	328, 239, 523	329, 484, 123	674,635, 6647
	**CMR ([95%CI]**	1.8	1.9	1.9	2	2	1.9	2	2	2	2	2.1	2.2	2.2	2.3	2.2	2.3	2.3	2.1	2.1	2.1	2.1	2.2	2.1
	**AAMR [95%CI}**	1.9	2	1.9	2	2	1.9	2	1.9	1.9	1.9	1.9	2	2	2.1	2	2	2	1.7	1.7	1.7	1.7	1.7	1.9
**Age**	**25–34 CMR**	0.1	0.0	0.0	0.1	0.1	0.0	0.0	0.0	0.0	0.1	0.0	0.0	0.1	0.1	0.0	0.0	0.0	0.0	0.1	0.0	0.0	0.0	
	**35–44** ** CMR**	0.4	0.4	0.4	0.4	0.4	0.4	0.4	0.4	0.4	0.4	0.3	0.4	0.4	0.3	0.3	0.4	0.3	0.3	0.3	0.3	0.3	0.3	
	**45–54** ** CMR**	1.2	1.4	1.2	1.2	1.2	1.3	1.3	1.3	1.4	1.4	1.5	1.4	1.4	1.5	1.4	1.4	1.4	1.3	1.4	1.4	1.4	1.3	
	**55–64** ** CMR**	3.3	3.9	3.6	4	4.1	3.7	3.8	3.7	3.5	3.7	3.9	4	3.9	4	3.9	4	3.8	3.9	4.1	3.7	3.9	3.8	
	**65–74 ** **CMR**	8.8	8.9	9	8.9	9	8.7	8.7	8.8	8.9	8.6	8.8	8.9	8.9	9.2	8.8	8.6	8.9	7.6	7.3	7.7	7.5	7.8	
	**75–84 ** **CMR**	11.8	12.3	12.3	12.8	12.6	11.6	12.4	12.2	12.4	12.2	11.6	12.4	12.9	13.3	12.5	12.7	12.6	10.2	10.3	10.1	10.2	10.5	
	**Over 85** ** CMR**	7.2	7.4	7	7.7	8.3	7.4	8	7.6	7.6	7.7	7.9	8	7.8	8.4	7.7	8.2	7.9	5.9	5.9	5.2	5.1	5.1	
**Sex**	**Number of ** **deaths**	2,702	2,906	2,935	2,980	3,110	3,077	3,197	3,287	3,204	3,335	3,455	3,579	3,670	3,920	3,870	4,006	4,063	3,701	3,791	3,808	3,888	4,007	
	**Male AAMR**	2.3	2.4	2.4	2.4	2.4	2.3	2.4	2.4	2.3	2.3	2.3	2.4	2.4	2.5	2.4	2.4	2.4	2.1	2.1	2	2	2.1	
	**Number of ** **deaths**	2425	2576	2463	2743	2758	2586	2675	2667	2869	2862	2918	3069	3179	3287	3174	3265	3340	3001	3071	3107	3154	3265	
	**Female AAMR**	1.6	1.6	1.5	1.7	1.7	1.5	1.6	1.6	1.6	1.6	1.6	1.7	1.7	1.7	1.6	1.6	1.6	1.4	1.4	1.4	1.4	1.4	
**Race**	**Number of ** **deaths**	4,809	5,137	5,042	5,349	5,452	5,271	5,480	5,549	5,643	5,768	5,896	6,107	6296	6,615	6,483	6,687	6,743	6,135	6,235	6,235	6,375	6,544	
	**White AAMR**	2.0	2.1	2.0	2.1	2.2	2.1	2.1	2.1	2.1	2.1	2.1	2.2	2.2	2.2	2.1	2.2	2.1	1.9	1.9	1.8	1.9	1.9	
	**Number of** ** deaths**	244	278	281	280	311	297	297	314	320	308	357	375	396	442	386	411	427	397	451	466	471	488	
	**Black or African** ** American** ** AAMR**	0.9	1.1	1.1	1.0	1.1	1.0	1.0	1.0	1.0	1.0	1.0	1.1	1.1	1.2	1.0	1.1	1.1	0.9	1.1	1.0	1.0	1.1	
	**Number of ** **deaths**	64	61	62	78	91	86	79	69	96	101	90	140	140	138	145	156	205	149	147	179	168	200	
	**Asian or Pacific ** **Islander AAMR**	0.9	0.8	0.8	0.9	1.0	0.9	0.7	0.6	0.8	0.8	0.7	1.1	1.0	0.9	0.9	0.9	1.1	0.8	0.7	0.8	0.8	0.9	
	**Number of ** **deaths**	10	<10	13	16	14	<10	16	22	14	20	30	26	17	12	30	17	28	21	29	35	28	40	
	**American** ** Indian or ** **Alaska Natives ** **AAMR**	Unreliable	Unreliable	Unreliable	Unreliable	Unreliable	Unreliable	Unreliable	0.9	Unreliable	0.7	1.3	1.0	Unreliable	Unreliable	1.0	Unreliable	0.8	0.6	0.8	0.9	0.7	0.9	

**Abbreviations:** AAMR – Age adjusted mortality rate; CI – Confidence interval; CMR – Crude mortality rate; PD – Parkinson’s Disease; SE – Standard error.

### Annual trends for MND-associated mortality related to age

**Figure 2.**
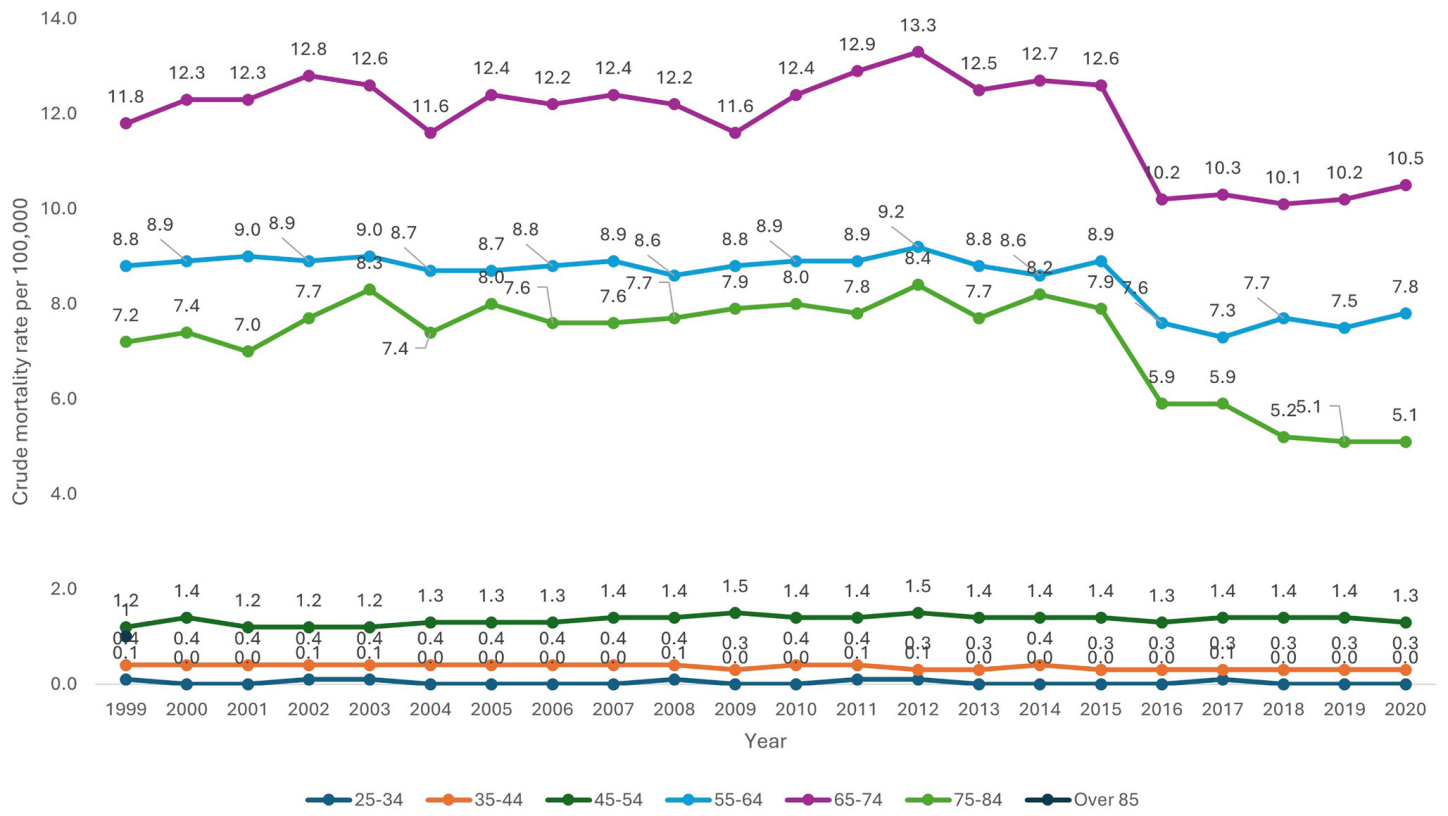
Crude mortality rate from 1999–2020 stratified by age.

Over the 20-year period, older age categories were associated with a consistently higher CMR. CMR for patients aged over 85 was 7.9 (95% CI 7.7-8.0), compared to a CMR of 1.5 (95% CI 1.4-1.5) for patients aged 45–54 ( Table 1 and Figure 2).

### Annual trends for MND-associated mortality stratified by sex

**Figure 3.**
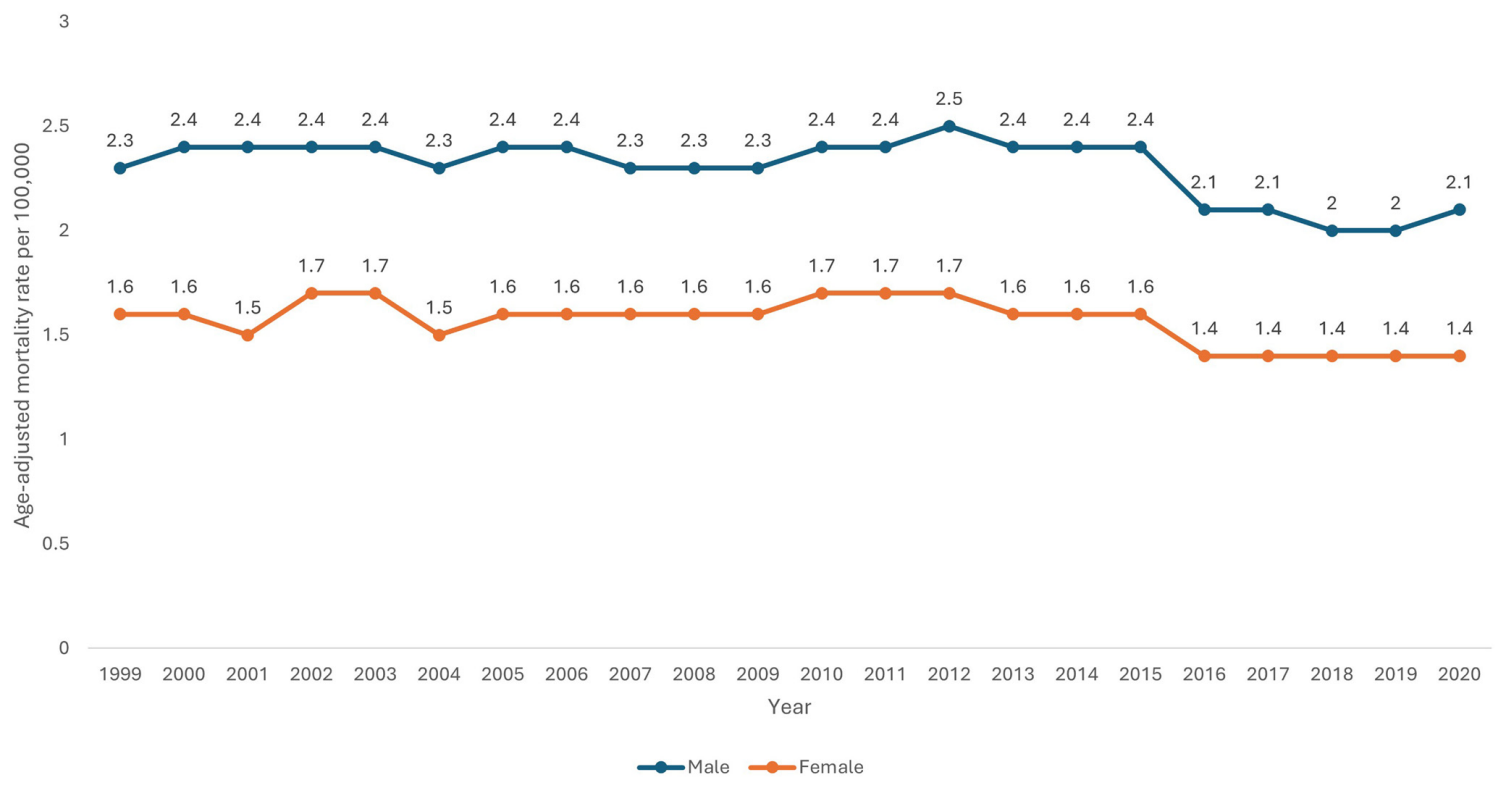
Age-adjusted mortality rate from 1999–2020 stratified by sex.

Male sex had a consistently higher AAMR (2.3 per 100,000 95% CI 2.3-2.3) than female sex (1.6 per 100,000 95% CI 1.5-1.6) across the study period. Using Joinpoint regression analysis, the APC in AAMR for males was 0.17 (95% CI 0.17-0.21) from 1999–2014, which then decreased to -4.75 (95% CI -4.75-0.24) from 2014–2018, and then increased to 2.54 (95% CI -2.64-5.84) from 2018–2020. The APC in AAMR for females was 0.39 (95% CI -0.19-1.48) from 1999–2012 which reduced to -2.57 (95% CI -4.98- -1.40) from 2012–2020 ( Table 1 and Figure 3).

### Annual trends for MND-associated mortality stratified by race/ethnicity

White patients had consistently higher AAMR (2.1 per 100,000 95% CI 2.0-2.1) than Black/African Americans (1.1 per 100,000 95% CI 1.0-1.1), American Indians/Alaska Natives (0.8 per 100,000 95% CI 0.7-0.9) and Asians/Pacific Islanders (0.8 per 100,000 95% CI 0.7-0.9). Using Joinpoint regression analysis, the APC in AAMR for white patients was 0.40 (95% CI -0.01-0.80) from 1999–2014, reducing to -4.73 (95% CI -7.48-0.48) from 2014–2018 and increasing to 3.39 (95% CI -2.42-7.16) from 2018 to 2020. Joinpoint regression for the Black/African American, Asians/Pacific Islander and American Indians/Alaska Native groups could not be calculated due to relatively small numbers across multiple years which CDC-WONDER assigns as unreliable.

### Annual trends for MND-associated mortality stratified by Latino status

**Figure 4.**
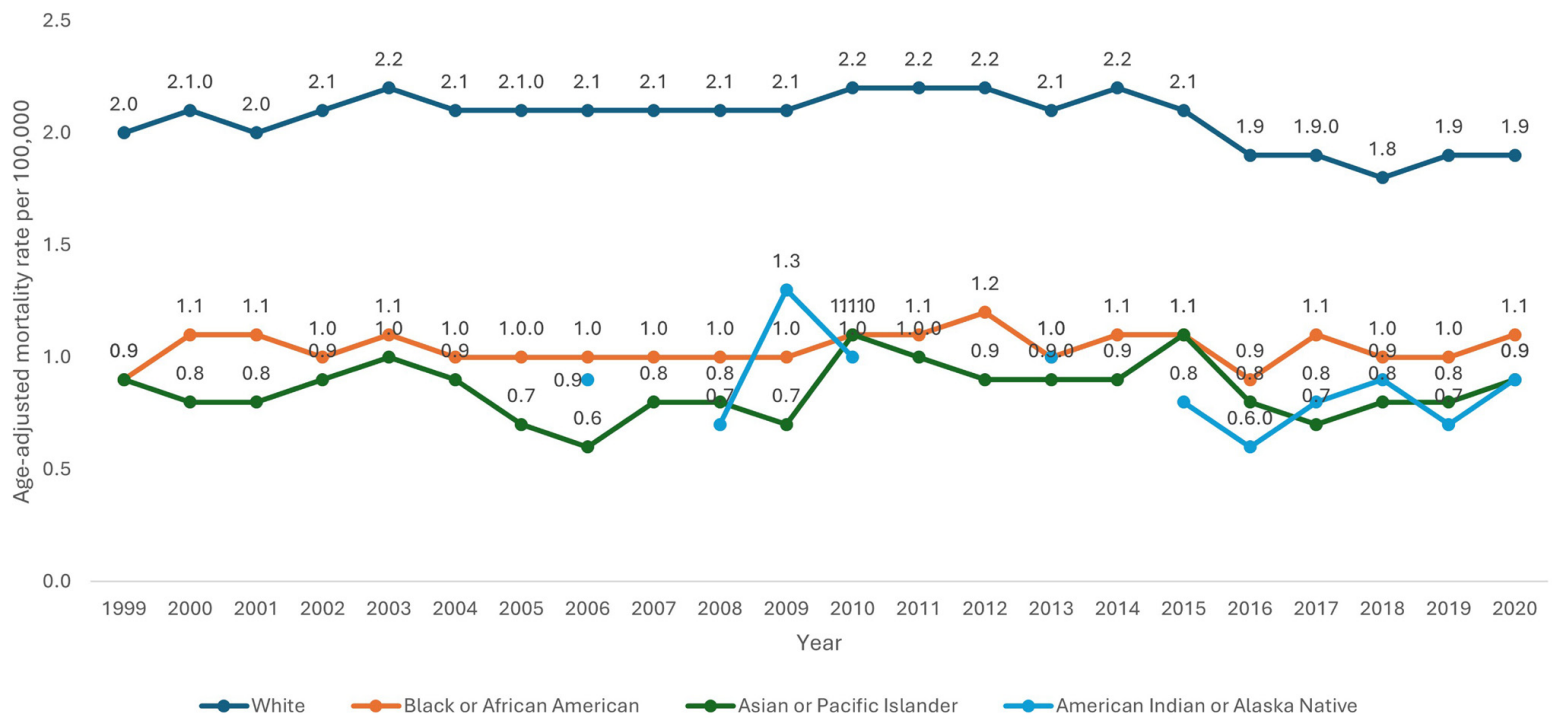
Age-adjusted mortality rate from 1999–2020 stratified by race.

 Overall AAMR across the study period was higher for non-Hispanic/Latino groups (AAMR 2.0 95% CI 2.0-2.0) than Hispanic/Latino groups (AAMR 1.1 95% CI 1.0-1.1) in the population. AAMR for Hispanic/Latino deaths was 0.09 per 100,000 (95% CI -0.36-0.55). On Joinpoint regression analysis, there were no discernible difference in trend for Hispanic/Latino groups than for the whole population. APC for AAMR for non-Hispanic/Latino deaths was 0.35 (95% CI -1.45-2.18) from 1999–2014, decreasing to -5.06 (95%CI -6.88-2.18) from 2014–2017, then increasing to 0.31 (95% CI -3.13-5.03) from 2017–2020 ( Table 1 and Figure 4).

### Census region and census division level data

The Midwest census region had the highest AAMR (2.1 per 100,000 95% CI 2.1-2.1), followed by the West (1.9 per 100,000 95% CI 1.9-2.0), Northeast (1.9 per 100,000 95% CI 1.8-1.9) and South (1.8 per 100,000 95% CI 1.8-1.9). The census division with the highest AAMR was West North Central (2.2 per 100,000 95%CI 2.2-2.3), followed by New England (2.2 per 100,000 95% CI 2.2-2.3), East North Central (2.1 per 100,000 95% CI 2.0-2.1), Mountain (2.0 per 100,000 Central (1.8 per 100,000 95% CI 1.8-1.8), Middle Atlantic 95% CI 2.0-2.0), Pacific (1.9 per 100,000 95% CI 1.8-1.9), (1.7 per 100,000 95% CI 1.7-1.7) and West South Central (1.7 per South Atlantic (1.8 per 100,000 95% CI 1.8-1.8), East South 100,000 95% CI 1.7-1.7) ( Table 2).

**Table 2.** Mortality rate stratified by Census region, Census division and HHS region.

	Variables	AAMR [95%CI]
**Census region**	**Census Region 1: Northeast**	1.9 [1.8-1.9]
	**Census Region 2: Midwest**	2.1 [2.1-2.1]
	**Census Region 3: South**	1.8 [1.8-1.8]
	**Census Region 4: West**	1.9 [1.9-2.0]
**Census division**	**Division 1: New England**	2.2 [2.2-2.3]
	**Division 2: Middle Atlantic**	1.7 [1.7-1.7]
	**Division 3: East North Central**	2.1 [2.0-2.1]
	**Division 4: West North Central**	2.3 [2.2-2.3]
	**Division 5: South Atlantic**	1.8 [1.8-1.8]
	**Division 6: East South Central**	1.8 [1.8-1.8]
	**Division 7: West South Central**	1.7 [1.7-1.7]
	**Division 8: Mountain**	2.0 [2.0-2.0]
	**Division 9: Pacific**	1.9 [1.8-1.9]
**HHS region**	**HHS Region #1**	2.2 [2.2-2.3]
	**HHS Region #2**	1.6 [1.6-1.6]
	**HHS Region #3**	2.0 [1.9-2.0]
	**HHS Region #4**	1.8 [1.7-1.8]
	**HHS Region #5**	2.1 [2.1-2.1]
	**HHS Region #6**	1.7 [1.7-1.8]
	**HHS Region #7**	2.1 [2.1-2.1]
	**HHS Region #8**	2.3 [2.2-2.4]
	**HHS Region #9**	1.7 [1.7-1.7]
	**HHS Region #10**	2.5 [2.4-2.5]
**State**	**Alabama**	1.7 [ 1.7 - 1.8 ]
	**Alaska**	1.8 [ 1.5 – 2.0 ]
	**Arizona**	1.9 [ 1.8 - 1.9 ]
	**Arkansas**	2.0 [ 1.9 - 2.1 ]
	**California**	1.7 [ 1.7 - 1.7 ]
	**Colorado**	2.3 [ 2.2 - 2.4 ]
	**Connecticut**	2.0 [ 2.0 - 2.1 ]
	**Delaware**	1.9 [ 1.8 - 2.1 ]
	**District of Columbia**	1.4 [ 1.2 - 1.6 ]
	**Florida**	1.6 [ 1.6 - 1.7 ]
	**Georgia**	1.7 [ 1.6 - 1.7 ]
	**Hawaii**	1.4 [ 1.3 - 1.5 ]
	**Idaho**	2.4 [ 2.3 - 2.6 ]
	**Illinois**	1.9 [ 1.8 - 1.9 ]
	**Indiana**	2.1 [ 2.0 - 2.2 ]
	**Iowa**	2.2 [ 2.1 - 2.3 ]
	**Kansas**	2.2 [ 2.1 - 2.3 ]
	**Kentucky**	1.8 [ 1.8 - 1.9 ]
	**Louisiana**	1.8 [ 1.7 - 1.8 ]
	**Maine**	2.5 [ 2.4 - 2.7 ]
	**Maryland**	1.9 [ 1.9 – 2.0 ]
	**Massachusetts**	2.2 [ 2.1 - 2.3 ]
	**Michigan**	2.1 [ 2.1 - 2.2 ]
	**Minnesota**	2.7 [ 2.6 - 2.8 ]
	**Mississippi**	1.6 [ 1.5 - 1.7 ]
	**Missouri**	2.0 [ 1.9 - 2.1 ]
	**Montana**	2.4 [ 2.2 - 2.6 ]
	**Nebraska**	2.1 [ 2.0 - 2.3 ]
	**Nevada**	1.4 [ 1.3 - 1.5 ]
	**New Hampshire**	2.2 [ 2.1 - 2.4 ]
	**New Jersey**	1.7 [ 1.6 - 1.7 ]
	**New Mexico**	1.7 [ 1.6 - 1.9 ]
	**New York**	1.5 [ 1.5 - 1.6 ]
	**North Carolina**	1.9 [ 1.9 – 2.0 ]
	**North Dakota**	2.1 [ 1.9 - 2.4 ]
	**Ohio**	2.1 [ 2.0 - 2.1 ]
	**Oklahoma**	2.0 [ 1.9 - 2.1 ]
	**Oregon**	2.5 [ 2.4 - 2.6 ]
	**Pennsylvania**	2.0 [ 1.9 - 2.0 ]
	**Rhode Island**	2.0 [ 1.8 - 2.2 ]
	**South Carolina**	1.9 [ 1.8 - 1.9 ]
	**South Dakota**	2.2 [ 2.0 - 2.4 ]
	**Tennessee**	1.9 [ 1.9 – 2.0 ]
	**Texas**	1.6 [ 1.6 - 1.7 ]
	**Utah**	2.3 [ 2.2 - 2.4 ]
	**Vermont**	2.8 [ 2.5 - 3.0 ]
	**Virginia**	2.0 [ 1.9 - 2.0 ]
	**Washington**	2.5 [ 2.4 - 2.6 ]
	**West Virginia**	2.0 [ 1.9 - 2.1 ]
	**Wisconsin**	2.4 [ 2.3 - 2.4 ]
	**Wyoming**	2.2 [ 2.0 - 2.5 ]

**Abbreviations:** AAMR – Age adjusted mortality rate; CI – Confidence interval; HHS – Health and Human Services.

### Health and Human Services region level data

The top 3 health and human services (HHS) regions with the highest AAMR was region 10 (Alaska, Idaho, Oregon, Washington) (2.5 per 100,000 95% CI 2.4-2.5), followed by region 8 (Colorado, Montana, North Dakota, South Dakota, Utah and Wyoming) (2.3 per 100,000 95% CI 2.2-2.4) and region 1 (Connecticut, Maine, Massachusetts, New Hampshire, Rhode Island, and Vermont) (2.2 per 100,000 95% CI 2.2-2.3) ( Table 2).

### State level mortality rate

The 3 states with the highest AAMR was Vermont (2.8 per 100,000 95% CI 2.5-3.0), followed by Minnesota (2.7 per 100,000 95% CI 2.6-2.8) and Maine (2.5 per 100,000 95% CI 2.4-2.7). The 3 states with the lowest AAMR over the study period was Nevada (1.4 per 100,000 95% CI 1.3-1.5), followed by the Hawaii (1.4 per 100,000 95% CI 1.3-1.5) and the District of Columbia (1.4 per 100,000 95% CI 1.2-1.6) ( Table 2).

### Urbanisation status

Small metro urbanisation had the highest overall AAMR (2.1 95% CI 2.0-2.1), followed by large fringe metro (AAMR 2.0 95% CI 2.0-2.0), medium metro (AAMR 2.0 95% CI 2.0-2.0), nonmetro micropolitan (AAMR 2.0 95% CI 2.0-2.0), nonmetro noncore (AAMR 1.9 95% CI 1.8-1.9) and large central metro (AAMR 1.6 95% CI 1.6-1.7) ( Table 2).

### Place of death

Of all patient deaths (140,945), the most common place of death was the decedent’s home (49.2%), followed by medical facility – inpatient (20.5%), nursing home/long term care (17.0%), hospice facility (5.7%), other place of death (4.5%), medical facility – outpatient/emergency room (2.5%), unknown place (0.3%) and medical facility – death on arrival (0.3%) ( Table 3).

**Table 3.** Location of death.

Location	Deaths	Percentage of Total Deaths, %
**Medical Facility - Inpatient**	28839	20.5%
**Medical Facility - Outpatient or ER**	3567	2.5%
**Medical Facility - Dead on Arrival**	415	0.3%
**Medical Facility - Status unknown**	81	0.1%
**Decedent's home**	69312	49.2%
**Hospice facility**	8049	5.7%
**Nursing home/long term care**	23990	17.0%
**Other**	6330	4.5%
**Place of death unknown**	362	0.3%

**Abbreviations:** ER – Emergency room.

## Discussion

### Summary of main findings

 This study investigated the demographic trends in MND-associated mortality using death certificate data from the US. There are several findings of note. First, age-adjusted MND-associated mortality decreased over a 20-year period, with 140,945 total deaths and AAMR was 1.9 per 100,000 in 1999 and 1.7 per 100,000 in 2020. Second, there were race and sex trends in MND-associated mortality, with male sex and white race associated with the highest AAMR consistently over the study period. Finally, there were notable geographical differences in MND-associated mortality. The region with the highest AAMR was the Midwest and the states with the highest AAMR were Vermont, Minnesota and Maine.

### MND and mortality

Over a 20-year period, we observed a rise in the deaths associated with MND. A US-based study from 2011–2014 found the overall age-adjusted mortality rate from MND was 1.70 per 100,000 (95% CI 1.68-1.72) ^19^. Additionally, a systematic analysis for the Global Burden of Disease found there was a 1.1% increase in the age standardised rates of years lived with disability (YLDs) amongst individuals with MND between 2006 and 2016 ^20^. This could be indicative of overall increase in life expectancy amongst affected patients from 1990 to 2016. Notably, Riluzole was approved by the FDA for the first pharmacological treatment of ALS in 1995 ^20^. The use of Riluzole for patients with motor neurone disease has been found to increase life expectancy, albeit by a modest 2–3 months of tracheostomy-free survival ^20^. A 2012 systematic review and meta- analysis of four randomised control trials concluded that use of Riluzole increased median survival from 11.8 to 14.8 months ^21^. Additionally, it is important to note that a multidisciplinary clinic approach also improves survival rate in ALS. One particular study in Ireland and northern Ireland showed that there was a survival benefit for patients who attended ALS clinics due to better community based care ^22^.

This analysis included 195 countries from 2006 to 2016, so may not be translatable solely to the pattern observed in the USA ^20^. Furthermore, increase in overall YLD across this timespan could be associated with improved access to healthcare for affected patients, and possibly earlier diagnosis.

### Sex differences in MND-mortality

We found there to be higher rates of MND associated mortality in males. Overall, male sex was associated with an AAMR of 2.3 per 100,000 (95% CI 2.3-2.3) whereas this figure for women in the same study period was 1.6 per 100,0000 (95% CI 1.5-1.6). This was concordant with findings from other studies ^3^. A UK study from 1990 to 2005, where age-standardised incidence was found to be 3.9 per 100,000 in men, in contrast to 2.6 per 100,000 in women ^23^. In this particular study, it was found that the incidence of MND was 54% higher in men than in women (95% CI: 33%-77%) ^23^. However, the trends in MND incidence have also been documented to vary over time in males and females. Whilst males were again found to experience a higher death rate than females ^22^, men over 65 also experienced a significant annual reduction in overall mortality from 1999 to 2009 (p<0.001). However, men aged 20–49 experienced an increasing death rate over the study period (p=0.01). The death rate for females showed no significant change over the period of the study, however, there was a general reduction in MND-associated mortality rates in persons over 65 years of age ^24^. The overall AAMR per 100,000 population was found to be higher in males (2.68) than in females (1.77) ^22^.

### Racial differences in MND-mortality

In this study, we consistently found higher rates of death in white patients, with an AAMR of 2.1 per 100,000 (95% CI 2.0-2.1). Black/African American patients experienced a lower MND-associated mortality rate of 1.1 per 100, 000 (CI 1.0-1.1), and both American Indians/ Alaska Natives and Asians/ Pacific Islanders had an AAMR of 0.8 per 100,000 (95% CI 0.7-0.9). Furthermore, the overall deaths of patients of non-white patients increase after 2010. This could be due to inadequate awareness of MND and it’s presentation in non-white groups. Therefore the time to diagnosis may be delayed, leading to worse outcomes if the disease is in a more advanced state ^25^. These findings are reflected in the literature. In a study investigating racial disparities in MND associated mortality from 1999 to 2006 in the University of Arkansas for Medical Sciences clinic for MND, 466 deaths were associated with MND. 95.5% of these patients were white, 3.6% were black, and 0.9% were from an ‘other’ category. At this time, the proportion of the Arkansas population who were black was 17% ^8^. However, the average age of onset for the black cohort was 52.8 ± 13.0 ( *p* = 0.038), whilst it was 58.1 ± 12.4 years for the white patients in this study. It is possible that genetic differences modify risk of the development of MND and maintenance of neurological function throughout the course of disease progression ^8^. However, it must also be considered that this disparity may, in part, be due to under-ascertainment of some cases due to poor access to healthcare as a result of socioeconomic deprivation ^8^. There was also a difference in physiological factors, such as average pre-morbid body mass index (BMI) between the group of black patients and white patients respectively. Black patients had a pre-morbid BMI of 31.2kg/m ^2^ (± 6.4) whilst white patients from this clinic had a premorbid BMI of 28.4 kg/m ^2 ^(± 5.3, *p* = 0.01). However, BMI also significantly reduced by the time of disease diagnosis in black patients compared to white patients ^8^.

Another study found no difference in the survival rates between affected black and white patients ^26^. This may be due to a multitude of factors, such as different sample sizes, study designs and variations in some health determinants such as socioeconomic status and availability of healthcare for more varied populations and racial groups across different states.

### Geographical differences in MND-mortality

A previous study has reported a strong correlation between MND-associated mortality rates and the proportion of the population that uses well water. It was hypothesised that this is due to well water serving as a reservoir for Legionella, which may be result in a pathophysiological process ending with central nervous system disease, and some cases of MND ^4^. This study was consistent in reporting Vermont as the state with the highest level of MND mortality, and cited Hawaii as the lowest ^4^. A significant association was observed between MND associated mortality and the proportion of the population within that region that uses water wells. The statistical model derived found that MND mortality increases by 0.00783 deaths per 100,000 people for every 1% rise in the population using well water ^4^.

It has consistently been documented that rates of MND associated mortality tend to be higher in US states that are situated at higher latitudes ^19^. It has also been found that countries at more northern latitudes in Europe experience a higher rate of ALS incidence. This could be due to multiple factors, including patterns of European settlement, genetic predisposition and environmental factors, and is in keeping with the highest recorded mortality rate being in Vermont, both in our findings and other literature, such as a study reporting all the causes of death in 2011–2014 coded under G12.2, which includes all motor neurone diseases including MND in the United States ^19^.

Beyond potentially causative environmental factors, multiple health determinants such as access to healthcare, genetic makeup of the district population and a multitude of environmental toxins as well as the genetic and epigenetic modulation of these may contribute to varying levels of both incidence of MND, rate of disease progression of MND, MND presentation and MND mortality. It is possible that the causative factor is associated with living in a northerly latitude ^19^ rather than solely ethnicity. However, ethnicity and latitude of habitation may be confounding factors in terms of MND incidence. Regardless, findings of differences in incidence between different ethnicities in less northerly latitudes can help to reflect that ethnicity may also play a role MND incidence, such as the findings in a study in Arkansas ^8^.

### Clinical implications

There are several clinical implications of this study. MND is an underlying cause of a significant number of deaths in the US. The knowledge of these trends, and the impact of specific demographics on mortality allows healthcare services to adapt to the needs of MND patients and identify individuals at greater risk of adverse outcomes early, allowing optimisation of their risk factors. Patients who are at greatest risk of adverse outcomes can be counselled on their risk and explore methods to mitigate modifiable risk factors. Furthermore, identifying disparities in care provides health services with the information they need to improve services and supports research into the underlying causes of such disparities. Furthermore, this study aids hypothesis building and the informing of other epidemiological and pathogenic studies regarding risk factors and causative factors. These may include genetic factors, ethnicity, and gender related predispositions as well as modifiable and environmental factors. This can be very useful in informing future clinical trials, and function as a crude pointer towards healthcare service utilisation.

### Limitations

There are several limitations to this study inherent to the dataset used. First, more granular analysis could not be conducted due to lack of data on pharmacotherapy, co-morbidities and procedures that could have mediated outcomes. This data could be used to provide adjusted analysis and comparisons between patient sub-groups to identify whether certain disparities exist after adjustment for these factors. Second, this data does not yield the exact cause of death. MND is recorded as a cause of death, but we cannot ascertain wither MND was the primary, secondary to even lower reason for death. Most MND related deaths are from respiratory complications, however the dataset does not provide the primary cause of death. Third, routinely collected electronic death certificate data is subject to information and selection bias, for instance due to differences in coding for causes of death across time, which could affect the internal validity of this study. Fourth, the dataset did not allow investigation into the subtypes of MND. MND subtypes have varying prognosis and therefore would have warranted further investigation. Finally, as CDC-WONDER provides information only on death-certificate data, analyses of quality of life and other aspects of MND care of interest to patients could not be investigated using these data.

### Future research

Future research could aim to reproduce the results of this research and improve it further through the use of adjusted analysis using a more detailed dataset to determine whether disparities in outcomes remain. Other research could explore why there is disparity in incidence and mortality rates across specific patient demographics. This could be across the translational pathway to determine whether there is a genetic susceptibility to the occurrence of MND and adverse events or complications from MND, or whether there are wider implications due to demographics such as social determinants of health that would be better addressed at the population level. Additionally, given the course of MND, a longitudinal study should aim to investigate the impact of these factors on survival duration.

## Conclusion

In conclusion, there are significant differences in trends in MND-related mortality in the US across demographic subgroups. Older age, male sex, white race, Midwest locality and Vermont, Minnesota and Maine state residence had the highest AAMR. The data obtained in this study is fundamental to build health services and palliative care pathways around the needs of this population group.

## Declarations

### Ethics and consent

This study was exempt from ethical approval from an institutional review board. CDC-WONDER is a publicly available, anonymised dataset created from routinely collected death certificate data and is covered by the provisions of the Public Health Service Act (42 U.S.C. 242m(d)).

## Data Availability

Under license by a third party and freely accessible at https://wonder.cdc.gov

## Software availability

Joinpoint Regression software is available to download for free at https://surveillance.cancer.gov/joinpoint/
