# Supplementary material for: Demographic trends of motor neurone disease-associated mortality from 1999-2020 in the United States
Source: NIHR Open Res. 2025 Aug 26;4:79. Originally published 2024 Dec 19. [Version 2] doi: 10.3310/nihropenres.13786.2 (PMC12673253; doi:10.3310/nihropenres.13786.2)
Supplement: Peer review history

# Reviewer report: Reviewer response for version 2

**DOI:** 10.3310/nihropenres.15199.r38416

**Published:** 2025-12-02

**Authors:** Dae-Gyu Jang

**Affiliations:** 1 University of Michigan, Ann Arbor, MI, USA

The authors investigated the demographic trends of MNDs using the available data. The authors may consider the following points to improve the article.

 1. There are several different methods to compute confidence intervals, such as normal CI for population mean, normal CI for population proportion, t-distribution CI. It is unsure that what method the authors used.

 2. In figure 1, it would be beneficial to present the 95% confidence band along with the fitter regression line.

 3. In discussion, the authors concluded that MND-associated mortality decreased from 1.9 to 1.7. Is this decrease statistically significant?

 4. Presenting numbers in a way such as 2.3 with 95% CI (2.3, 2.3) may not provide detailed information. The authors may consider providing more digits. For example, 2.314 with 95% CI (2.311, 2.325).

 5. While figure 1 visually explains that join point model may work better than simple linear regression, it is unsure that this model fits better to the data, compared to alternative methods. It may be worth comparing join point model with others, such as simple linear regression or piecewise regression models.

 6. While method section says "log-linear models for temporal variations.", the fitted lines in figure 1 look linear rather than log-linear. Did the authors use both linear and log-linear models in this study? It may be worth providing more information to clarify it.

Is the work clearly and accurately presented and does it cite the current literature?

Yes

If applicable, is the statistical analysis and its interpretation appropriate?

Partly

Are all the source data underlying the results available to ensure full reproducibility?

Yes

Is the study design appropriate and is the work technically sound?

Yes

Are the conclusions drawn adequately supported by the results?

Yes

Are sufficient details of methods and analysis provided to allow replication by others?

Yes

Reviewer Expertise:

Biostatistics, and Epidemiology

I confirm that I have read this submission and believe that I have an appropriate level of expertise to confirm that it is of an acceptable scientific standard.

# Reviewer report: Reviewer response for version 2

**DOI:** 10.3310/nihropenres.15199.r37017

**Published:** 2025-10-03

**Authors:** Stella Lischewski

**Affiliations:** 1 Universitätsklinikum Aachen, RWTH Aachen, Aachen, Deutschland, Germany

Many thanks for your comments on my suggestions. I still have reservations before I can recommend this manuscript for indexing:

 Comment 2: I still disagree with the use of the term race, even if this term is used in the original database, but this suggestion will not affect my recommendation for publication.

 Comment 3: My suggestion was to provide data on age, sex, ethnicity etc. but you specify in the limitation section that data on pharmacotherapy, comorbidities and outcomes are not available, this is not the same. Are these demographic data also not available in CDC Wonder? If so, I would suggest to amend the limitations section or, otherwise, include these data.

 Comment 5: I am aware that all-cause mortality is very heterogenous, but this comparison would help the reader gain a sense of whether the increased mortality in males or specific locations is a general trend in the US or specific to MND. There is no need to "correlate" this with the study findings, just a descriptive comparison in the discussion, and presumably these figures are available and do not need to be calculated. I do not understand why this could not be done.

 Comment 7: This misunderstanding has arisen because it is not clear that the "-" of the lower confidence interval is a minus sign and the "-" of the upper confidence interval is a hyphen, it looks like to negative numbers (in which case 0 would not have been included in the confidence interval). Best to use comma to separate confidence intervals.

Is the work clearly and accurately presented and does it cite the current literature?

Yes

If applicable, is the statistical analysis and its interpretation appropriate?

Partly

Are all the source data underlying the results available to ensure full reproducibility?

No source data required

Is the study design appropriate and is the work technically sound?

Partly

Are the conclusions drawn adequately supported by the results?

Yes

Are sufficient details of methods and analysis provided to allow replication by others?

Partly

Reviewer Expertise:

Neurodegeneration, Ataxia, previously NMD

I confirm that I have read this submission and believe that I have an appropriate level of expertise to confirm that it is of an acceptable scientific standard, however I have significant reservations, as outlined above.

# Reviewer report: Reviewer response for version 2

**DOI:** 10.3310/nihropenres.15199.r37016

**Published:** 2025-09-07

**Authors:** Jeannine M Heckmann

**Affiliations:** 1 University of Cape Town, Rondebosch, Western Cape, South Africa

the authors have satisfactorily attended to my comments

Is the work clearly and accurately presented and does it cite the current literature?

Yes

If applicable, is the statistical analysis and its interpretation appropriate?

I cannot comment. A qualified statistician is required.

Are all the source data underlying the results available to ensure full reproducibility?

Yes

Is the study design appropriate and is the work technically sound?

Yes

Are the conclusions drawn adequately supported by the results?

Partly

Are sufficient details of methods and analysis provided to allow replication by others?

Yes

Reviewer Expertise:

Neuromuscular disease, including ALS, in Africa

I confirm that I have read this submission and believe that I have an appropriate level of expertise to confirm that it is of an acceptable scientific standard.

# Reviewer report: Reviewer response for version 1

**DOI:** 10.3310/nihropenres.14974.r34231

**Published:** 2025-01-10

**Authors:** Stella Lischewski

**Affiliations:** 1 Universitätsklinikum Aachen, RWTH Aachen, Aachen, Deutschland, Germany

In this manuscript, Imantalab and colleagues investigated trends in MND-associated mortality using death certificate data from the public US-based CDC-Wonder registry. The authors conclude that older age, male sex, white ethnicity, and Midwest locality were associated with the highest AAMR. Overall, this work represents a valuable addition to the literature, particularly given the large size of the registry. However, I have several reservations and suggestions for revisions:

1. **Inclusive Language**:
  - The term "race" should be reconsidered to align with inclusive language practices (see Naini et al., *The Lancet*, 2024) [ref-1]. Additionally, "Hispanic status" or "Latino status" may not be sufficiently inclusive and could be rephrased to reflect modern terminology regarding ethnicity and cultural identity.
2. **Cohort Description**:
  - A more thorough description of the study cohort would enhance the manuscript. Providing data on the means and proportions of baseline characteristics (e.g., mean age, sex, ethnicity, and geographic location) for individuals who died of MND would offer readers a clearer understanding beyond AAMR and CMR values.
3. **AAMR Calculation**:
  - Clarify how AAMRs stratified by sex, ethnicity, and geographic region were calculated. For instance, if AAMRs are derived from dividing the number of deaths by the respective total population subgroup, it would be important to report the population sizes used in these calculations as per previous point.
4. **Contextual Comparison**:
  - The findings regarding increased mortality rates in males, specific locations, etc., would greatly benefit from a comparison with all-cause mortality rates in the US e.g. using previous literature.
5. **Table Formatting**:
  - The population data in Table 1 is difficult to read due to the absence of separators in large numbers. I recommend using commas (e.g., 279,040,168) to enhance readability.
6. **Annual Trends**:
  - In the section "Annual Trends for MND-associated Mortality," the reported APC for AAMR (0.30, 95% CI -2.81 to 5.94) appears inconsistent with its confidence intervals. Please clarify or address this discrepancy, as it may indicate a reporting or calculation error.

Is the work clearly and accurately presented and does it cite the current literature?

Yes

If applicable, is the statistical analysis and its interpretation appropriate?

Partly

Are all the source data underlying the results available to ensure full reproducibility?

No source data required

Is the study design appropriate and is the work technically sound?

Partly

Are the conclusions drawn adequately supported by the results?

Yes

Are sufficient details of methods and analysis provided to allow replication by others?

Partly

Reviewer Expertise:

Neurodegeneration, Ataxia, previously NMD

I confirm that I have read this submission and believe that I have an appropriate level of expertise to confirm that it is of an acceptable scientific standard, however I have significant reservations, as outlined above.

# Response

**Published:** 2025-05-06

**Authors:** Balamrit Singh Sokhal

**Reviewer's Comments (2)**

**Comment 1:** **In this manuscript, Imantalab and colleagues investigated trends in MND-associated mortality using death certificate data from the public US-based CDC-Wonder registry. The authors conclude that older age, male sex, white ethnicity, and Midwest locality were associated with the highest AAMR. Overall, this work represents a valuable addition to the literature, particularly given the large size of the registry. However, I have several reservations and suggestions for revisions.**

**Response: **We thank the reviewer for their favourable account of this manuscript and the opportunity to improve it further. We provide a point-by-point to reviewer comments below.

**Comment 2:** **The term "race" should be reconsidered to align with inclusive language practices (see Naini et al., *The Lancet*, 2024) [ref-1]. Additionally, "Hispanic status" or "Latino status" may not be sufficiently inclusive and could be rephrased to reflect modern terminology regarding ethnicity and cultural identity.**

**Reponse:** Thank you for your comment. Whilst, the reference noted is important to guide nomenclature for future academic practice, the terms used in the paper we have provided retains the terms used in the primary data source (CDC WONDER). We have sought not to change this as data were coded on the original, publicly available dataset using these terms. We hope this apporpirately addresses this comment.

**Comment 3:** **A more thorough description of the study cohort would enhance the manuscript. Providing data on the means and proportions of baseline characteristics (e.g., mean age, sex, ethnicity, and geographic location) for individuals who died of MND would offer readers a clearer understanding beyond AAMR and CMR values.**

**Response: **Thank you for your comment. We agree that a more granular analysis of individual level data could provide more rich data and a clearer understanding behind the analysis. Unfortunately, CDC WONDER provides data at the state, region and population level. This database does not provide data at the individual level. We have specified this in the limitations section of the manuscript.

*Under ‘Limitations’ section: “First, more granular analysis could not be conducted due to lack of data on pharmacotherapy, co-morbidities and procedures that could have mediated outcomes. This data could be used to provide adjusted analysis and comparisons between patient sub-groups to identify whether certain disparities exist after adjustment for these factors.”*

**Comment 4:** **Clarify how AAMRs stratified by sex, ethnicity, and geographic region were calculated. For instance, if AAMRs are derived from dividing the number of deaths by the respective total population subgroup, it would be important to report the population sizes used in these calculations as per previous point.**

**Response: **We thank the reviewer for their comment. AAMR are calculated by dividing the number of deaths by the population, including by subgroup. Crude number of deaths have been reported in the tables of the manuscript.

*Under ‘Tables’ section.*

**Comment 5:** **The findings regarding increased mortality rates in males, specific locations, etc., would greatly benefit from a comparison with all-cause mortality rates in the US e.g. using previous literature.**

**Response: **We thank the reviewer for their comment. This study sought to investigate the trends in mortality from MND stratified by various demographics over the 20 year inclusion period. We agree that an investigation of all-cause mortality stratified by patient demography would be interesting. However, causes of mortality are heterogenous and may be difficult to correlate to the findings of our study of specifically MND, therefore comparisons to all-cause mortality are beyond the scope of our study and have not been included. We hope the reviewer understands the reasons why comparisons with on all-cause mortality were not elaborated on further in the manuscript.

**Comment 6:** **The population data in Table 1 is difficult to read due to the absence of separators in large numbers. I recommend using commas (e.g., 279,040,168) to enhance readability.**

**Response: **We thank the reviewer for their comment. We have amended the tables anf gures to make them easier to read as suggested.

**Comment 7:** **In the section "Annual Trends for MND-associated Mortality," the reported APC for AAMR (0.30, 95% CI -2.81 to 5.94) appears inconsistent with its confidence intervals. Please clarify or address this discrepancy, as it may indicate a reporting or calculation error.**

**Response: **Thank you for your comment. We have verified that the APC in AAMR from 1999-2014 was 0.30% (95%CI -.2.81-5.94). Due to the confidence intervals crossing the zero value, this result was not statistically significant. Therefore we can reasonably conclude that the AAMR from 1999-2014 remained stable, and there was no significant change for this time period. We hope this adequately addresses the comment.

# Reviewer report: Reviewer response for version 1

**DOI:** 10.3310/nihropenres.14974.r34285

**Published:** 2025-01-03

**Authors:** Jeannine M Heckmann

**Affiliations:** 1 University of Cape Town, Rondebosch, Western Cape, South Africa

This audit of the CDC database for mortality stats in the US related to ALS has a main objective to determine where the gaps in health services are for patients with ALS. The report is well written. The conclusion of the work that ALS mortality is higher in older white males is somewhat circular since the authors acknowledge that there are many limitations to access of other races to healthcare. The main limitation being that a patient with poor access to health services may not receive a diagnosis of ALS and therefore will never be recorded as dying from ALS. This would be the very group which would need better services. To this end I feel the obvious gap in the work is that the granularity of the place where patients die, may offer a glimpse to this socioeconomic problem. For example, those patients who are offered a timeous diagnosis and are prepared for palliative care will likely die at home or in hospice. What is the racial makeup of these patients and the ones that die in ERs and in hospital and other?

 I suggest less emphasis on riluzole and other "therapies" (3 month survival increase) and the possibility that the reduced mortality  >2015 could be due to better clinics? (since none of the treatment trials have been positive excl SOD1 cases).

 Also, why did the deaths in Blacks and other races double after 2010 compared to <2010? increased awareness of ALS?

Is the work clearly and accurately presented and does it cite the current literature?

Yes

If applicable, is the statistical analysis and its interpretation appropriate?

I cannot comment. A qualified statistician is required.

Are all the source data underlying the results available to ensure full reproducibility?

Yes

Is the study design appropriate and is the work technically sound?

Yes

Are the conclusions drawn adequately supported by the results?

Partly

Are sufficient details of methods and analysis provided to allow replication by others?

Yes

Reviewer Expertise:

Neuromuscular disease, including ALS, in Africa

I confirm that I have read this submission and believe that I have an appropriate level of expertise to confirm that it is of an acceptable scientific standard, however I have significant reservations, as outlined above.

# Response

**Published:** 2025-05-06

**Authors:** Balamrit Singh Sokhal

**Reviewer's Comments (1)**

**Comment 1:**

 This audit of the CDC database for mortality stats in the US related to ALS has a main objective to determine where the gaps in health services are for patients with ALS. The report is well written. The conclusion of the work that ALS mortality is higher in older white males is somewhat circular since the authors acknowledge that there are many limitations to access of other races to healthcare. The main limitation being that a patient with poor access to health services may not receive a diagnosis of ALS and therefore will never be recorded as dying from ALS. This would be the very group which would need better services. To this end I feel the obvious gap in the work is that the granularity of the place where patients die, may offer a glimpse to this socioeconomic problem. For example, those patients who are offered a timeous diagnosis and are prepared for palliative care will likely die at home or in hospice. What is the racial makeup of these patients and the ones that die in ERs and in hospital and other?

**Response:**

 We thank the reviewer for their comment and positive appraisal of the manuscript. Access to healthcare is an important issue and can mediate outcomes. As requested, we have provided a supplementary table to compliment the main analyses by stratifying location of death by sex and race. Many of these numbers were small, especially for non-white and non-black populations, therefore, analysis by year could not be performed. Furthermore, due to availability of data and low numbers in the CDC WONDER database, analysis of percentage of deaths as a proportion of total number of deaths in the demographic were not able to be performed. We hope this adequately addresses the comment.

**Comment 2:**

 I suggest less emphasis on riluzole and other "therapies" (3 month survival increase) and the possibility that the reduced mortality  >2015 could be due to better clinics? (since none of the treatment trials have been positive excl SOD1 cases).

**Response:**

 Thank you for your comment. The observed trends could be due several reasons. One of which could be due to better patient follow up in clinics via a multidisciplinary approach. In particular this improves better patient care in clinics. Therefore, this has been noted in our study. Additionally, we believe it is also important to highlight the potential impact newly approved medical treatments that could aid with the quality of life of patients with MND.

**Comment 3:**

 Also, why did the deaths in Blacks and other races double after 2010 compared to <2010? increased awareness of ALS?

**Response:**

 Thank you for your comment. It is clear that there are disparities in the race of the patient and the clinical outcomes. The overall deaths of patients of non-white ethnicities increase after 2010. This could be due to better awareness of ALS presentation differing between the various races. Therefore the onset of diagnosis itself may be delayed, leading to worse outcomes. We have included this in the manuscript.

*Under 'Discussion' section: “F* *urthermore, the overall deaths of patients of non-white patients increase after 2010. This could be due to inadequate awareness of MND and its presentation in non-white groups. Therefore the time to diagnosis may be delayed, leading to worse outcomes if the disease is in a more advanced state.”*

*Under 'Discussion' section:* *“Additionally, it is important to note that a multidisciplinary clinic approach also improves survival rate in ALS. One particular study in Ireland and northern Ireland showed that there was a survival benefit for patients who attended ALS clinics due to better community based care.”*
